# Carbon-Based Nanomaterials in Biomass-Based Fuel-Fed Fuel Cells

**DOI:** 10.3390/s17112587

**Published:** 2017-11-10

**Authors:** Le Quynh Hoa, Mun’delanji C. Vestergaard, Eiichi Tamiya

**Affiliations:** 1 Federal Institute for Materials Research and Testing (BAM), Unter den Eichen 87, Berlin 12205, Germany; 2 Department of Biochemical Science and Technology, Faculty of Agriculture, Kagoshima University, Kagoshima 890-0065, Japan; 3 Department of Applied Physics, Osaka University, 2-1 Yamadaoka, Suita, Osaka 565-0871, Japan; tamiya@ap.eng.osaka-u.ac.jp

**Keywords:** carbon-based nanomaterials, biofuel cells, biomass, carbon nanotubes, graphene, carbon nanodots

## Abstract

Environmental and sustainable economical concerns are generating a growing interest in biofuels predominantly produced from biomass. It would be ideal if an energy conversion device could directly extract energy from a sustainable energy resource such as biomass. Unfortunately, up to now, such a direct conversion device produces insufficient power to meet the demand of practical applications. To realize the future of biofuel-fed fuel cells as a green energy conversion device, efforts have been devoted to the development of carbon-based nanomaterials with tunable electronic and surface characteristics to act as efficient metal-free electrocatalysts and/or as supporting matrix for metal-based electrocatalysts. We present here a mini review on the recent advances in carbon-based catalysts for each type of biofuel-fed/biofuel cells that directly/indirectly extract energy from biomass resources, and discuss the challenges and perspectives in this developing field.

## 1. Introduction

### 1.1. From Biomass to Biofuels: Conventional Processes and Challenges

Biomass, a renewable source of fuel made from biological materials such as plants and animal waste, has been used as the primary energy source since ancient times. In particular wood serves as the principle energy source till the dawn of the industrial revolution when fossil fuels became the absolute dominator. Besides a lot of advantages, the reproduction of fossil fuels is impossible making it a deathly Achilles heel compared to wood crops, the most widely used biomass resource, which can re-grow every 50–100 years. Moreover, fossil fuel reservoirs are going to emptying and its related environmental problems are severe. Therefore reducing dependence on fossil fuels becomes a more and more ultimate request. One of current solutions is to transform a variety of agricultural crops and their byproducts into a supplementary energy source. 

Currently, to extract energy, woody plants and grasses are used in several ways: The first is the same as our accentors did a hundred years ago which is to burn woody material and grasses to provide heat and/or steam for households or for generating electric power. This approach is still widely used in many underdeveloped countries. The second is to thermally decompose biomass under varied conditions such as pressure, temperature and catalyst to obtain combustible products. Another way is to ferment carbohydrates and then distil them to obtain ethanol, a highly ranked liquid fuel that can be blended with gasoline for motor vehicle use. Last but not the least, is the use of an anaerobic digestion processes in which microorganisms breakdown biomass in the absence of oxygen to produce biogas (mainly CO_2_ and CH_4_). These gaseous fuels can be further purified in an upgrader system to more desirable forms that are suitable for engines, gas turbines, fuel cells, boilers, industrial heaters, etc. albeit at some loss of energy [1,2,3,4,5]. Manufacturing combustible fuels from biomass inevitably involves intensive energy processes such as drying, pulverizing to minimize heat-transfer resistance and residue removal which not only results in partial loss of total heating value but also increases the cost of the conversion process. Consequently, researchers are looking for an optimum breakdown process coupled with effective energy conversion devices, which can gain a positive net energy and at the same time have low to acceptable costs. To that end, biomass-based electricity could be an appropriate answer. 

**Figure 1:**
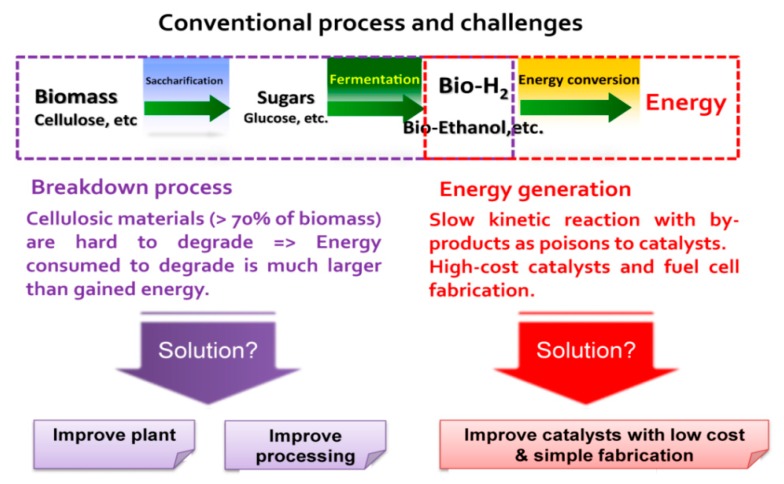
The conventional process and challenges in converting biomass sources into biofuels and ultimately generate energy in form of electricity.

As shown in Figure 1, the convention process to generate energy from biomass, especially electricity, consists of three main processes: (1) scarification (or breakdown process) to convert biomass to smaller components that are easier to be degraded; (2) fermentation to convert these small components to bio-fuels; and (3) energy conversion process that generates electricity from bio-fuels using fuel cells. In order to vastly improve overall efficiency, the scientific portfolio is organized into three core strategies: (1) improve plants; (2) improve processing; and (3) improve catalysts, targeting the three main processes, respectively [6,7,8,9,10,11,12,13,14,15,16,17,18,19,20]. Ideally, crude and abundant fuel sources such as cellulose or starch could be utilized directly [15]. However, despite the enormous efforts by scientists worldwide, this goal seems too far from real practical implementation. Thus, researchers aim to increase energy density and the degradability of plants such as grasses and other non-traditional oil crops by understanding, and manipulating metabolic flux and genetic modification [6,7]. Another solution is to improve the breakdown and fermentation processes in which the primitive biomass sources are degraded into smaller sources such as glucose and/or other monosaccharides, and ultimately to bio-hydrogen and bio-ethanol [8,9,10,11,12,13,14]. This research trend is focusing on the development of genetically modified microorganisms and efficient low-cost enzymes. Especially, bio-hydrogen and cellulosic ethanol-producing microbes are at the center of this strategy. After breaking down biomass sources into biofuels, the last challenge is utilizing as much as possible, the energy stored in the chemical bonds by energy conversion devices-fuel cells. At this stage, nanotechnology is being employed to improve cell performance, increase durability, and reduce the cost [16]. For example, precious metal electrocatalysts used are reduced to the nanoscale, ensuring a high catalytic surface area and minimizing the amount of precious metal used to maintain high performance. Thus far, many researchers have been studying inorganic nanostructured catalysts including alloys, bimetallic, and ternary metallics in order to not only reduce the noble metal loading but also create synergistic effects to enhance catalytic performance as well as durability [17]. Differing from the inorganic approach, a new strategy is focusing on organic-based nanomaterials, mostly carbon-based, as supporting matrices to fine-tune the catalytic activities of low-loading noble or non-noble metals toward desired products [18]. Graphene, for instance, when coated with cobalt and cobalt-oxide nanoparticles, is reported to be able to catalyze the oxygen reduction reaction on the cathode side of fuel cells, nearly as well as platinum does and is substantially more durable [19]. A catalyst made of iron nanoparticles confined inside pea-pod-like carbon nanotubes exhibits a high activity and remarkable stability as a cathode catalyst in polymer electrolyte membrane fuel cells [20]. 

In this mini-review, we discuss the main results from recent studies on fuel cells that directly or indirectly utilized biomass as fuels and carbon-based nanomaterials as catalytic materials. From the discussion, the challenges will be made more succinct, exposing the future prospects of this research trend. 

### 1.2. Biofuel-Fed Fuel Cells and Biofuel Cells

**Figure 2:**
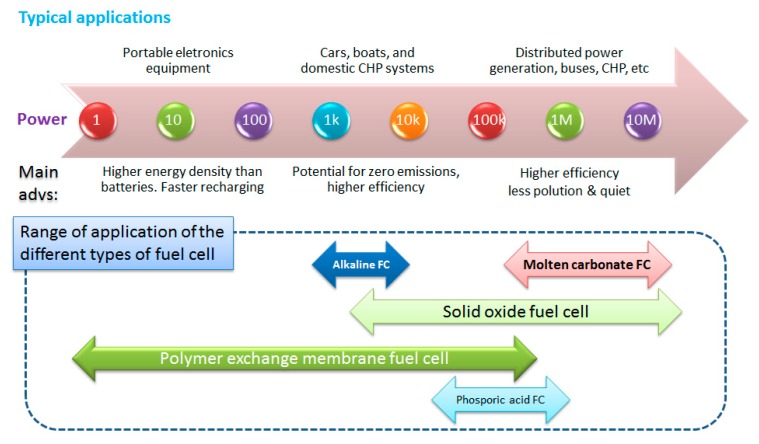
Summary of applications and main advantages of different types fuel cells (CHP stands for combined heat and power systems. The power unit is Watt).

The first electrochemical cell (fuel cell) akin to the Volta cell (a battery) was discovered in 1839 by Sir William R. Grove [21]. A fuel cell is an electrochemical “device” that continuously converts chemical energy into electric energy (and some heat) for as long as fuel and oxidants are supplied. A fuel cell shares similar electrochemical properties with a battery. However, it does not need recharging, operates quietly and efficiently. A fuel cell also is considered as an ideal substitution for combustion engines in the near future. Thermodynamically, the most striking difference is that thermal engines are limited by the Carnot efficiency while fuel cells are not. The limiting factors are the temperature at which the heat enters the engine, *T_H_*, and the temperature of the environment into which the engine exhausts its waste heat, *T_C_*. No device converting heat into mechanical energy, regardless of its construction, can exceed the Carnot cycle efficiency: η ≤ 1 − *T_C_*/*T_H_*. In contrast, since all the components in fuel cell systems work at the same temperature (*T = T_H_ = T_C_*) it is clearly not limited by the Carnot’s theorem. This is because the Carnot’s theorem applies to engines converting thermal energy into work, whereas fuel cells convert chemical energy into work. Therefore, the energy conversion efficiency can reach more than 50% and can be enhanced by stacking single fuel cells and utilizing a vast range of fuel sources such as hydrogen, natural gas, methanol, and ethanol. Figure 2 shows the summary of the power ranges and applications of different types of fuel cells [21,22,23]. However, the problem is that even with hydrogen fuel cells, the most successful proton exchange membrane fuel cell (PEMFC) system, there are huge challenges including fuel distribution and safety issues; and methanol of direct methanol fuel cell (DMFC) is toxic. In addition, the fuel production is not without concern. It has its own print in greenhouse gas emission and climate change. Today 96% of all hydrogen is derived from fossil fuels, with 48% from natural gas, 30% from hydrocarbons, 18% from coal, and about 4% from electrolysis [24]. The situation is similar for methanol production since it is produced by steam-methane reforming from natural gas source. Thus, researchers have been pushed to search for more practical, safer and “greener” solution(s): biomass derived-biofuels. For biofuel cells, it is not necessary to use biomass as fuel. “Bio”-fuel cells use biocatalytic systems such as enzymes or microorganisms as catalysts. In the frame of green-chemistry, this review discusses the applications of CBNs for biomass-based fuel-fed fuel cells, which include biofuel cells and do not exclude the fuel cells that utilize non-biocatalytical systems. 

### 1.3. Carbon-Based Nanomaterials

**Figure 3:**
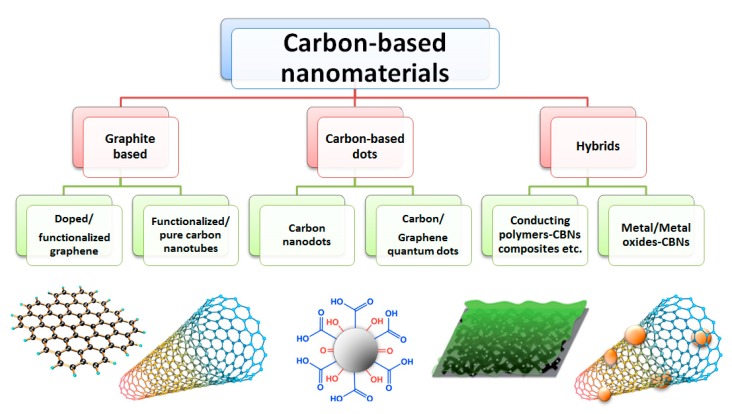
Carbon-based nanomaterials that have been and have potential to be used for biofuel-fed fuel cells.

Carbon-based nanomaterials (CBNs), were discovered about 150 years ago, remarkably in 1991 by a physicist, Sumio Iijima of Nippon Electric Company (NEC) Corporation, Japan, who published a ground-breaking paper in Nature on multiwalled carbon nanotubes (CNTs) [25], and in 2004 by Novoselov with the mechanical exfoliation of graphite to produce the first single layer graphene [26]. They have since been thoroughly characterized, and extensively developed for a wide range of fields from industrial applications such as high-strength materials, nanoscale electronic, high efficiency electron emitters, to research including biosensors, drug delivery systems, and tissue scaffold reinforcement [27]. CBNs have enjoyed this wide application due to their unique combinations of mechanical, optical, thermal, and electrical properties. Graphene, for example, is incredibly strong with the tensile strength of ~130 GPa, for a defect free single layer, Young’s modulus of 1 Tpa, third order elastic stiffness ≈ 2 Tpa; and at the same time is very flexible and light (0.77 mg m^−2^) with a very high specific surface area (~2630 m^2^ g^−1^) [28]. Figure 3 shows the CBNs that have been significantly used and can potentially be used for biofuel-fed fuel cells.

One of the CBNs characteristics, which is important in fuel cell applications, is high electronic conductivity. In the past, this could be achieved by amorphous carbon, which possesses not only good conductivity but also large surface area and porosity. With CBNs, not only is the conductivity much more increased, but also the weight and size of electrode could be extremely reduced. For instance, electronic transport in carbon nanotubes ballistically occurs over long nanotube lengths due to their nearly one-dimensional structure, thus enabling them to carry high current without heating effect. When employed as electrode materials for fuel cells, a network of CNTs as catalytic supports not only enhances the active surface area, mass transport of fuels, conductivity, and much more resistance to corrosion but also is involved in the catalytic mechanism (with or without precious metals) toward desired products with faster reaction kinetics, leading to higher power density and energy conversion efficiency [29,30,31,32,33,34]. Comparable and higher catalytic effects compared to that of traditional Pt/C have been also observed in the case of graphene doped/co-doped with various heteroatoms and their composites with metal/metal oxides and/or conducting polymers. Table 1 provides a summary of the most recent achievemenst of CBNs, from 2010 (top) to 2017 (bottom), in cathodic catalytic systems that deal with oxygen reduction reaction, a main limiting factor to the cell performance, especially in the case of microbial fuel cells (MFCs). Further applications of carbon-based nanomaterials in anodic catalytic systems, which deals with oxidation reactions of biomass-based fuels are discussed in the coming sections. 

## 2. Direct Energy Conversion Challenges and Applications of Carbon-Based Nanomaterials

### 2.1. Cellulose and Cellulosic Biomass Fuel Cells

**Figure 4:**
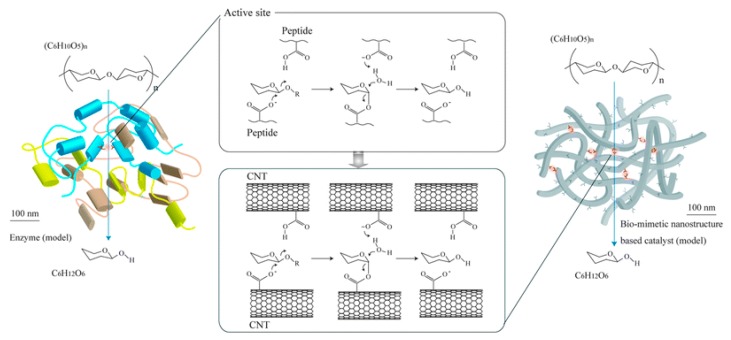
Proposed biomimetic functionalized carbon nanotubes-based catalyst for direct breaking down of cellulose. Reprinted with permission from ref. [49]. Copyright 2011, RSC.

Since more than 70% of biomass is made of cellulose, the most obvious biomass target for biofuel-fed fuel cells is cellulose. However, cellulose, a straight chain natural polymer (C_6_H_10_O_5_)_n_, is insoluble in water and as well as most other organic solvents, must first be hydrolyzed to a soluble substrate that can be oxidized on the surface of anode electrode or to be taken up by the cell. Sugano et al. succeeded in dissolving pure cellulose powder in alkaline solution using a freezing/thawing process and oxidizing it on gold electrode as anode, producing the first direct cellulose fuel cell with power density of 44 mW m^−2^ (150 mV and ~450 mA m^−2^) [15]. Recognizing the high-cost and active surface limitation of the gold-based electrodes, further research was done by the same group. They utilized functionalized carbon nanotubes as catalytic system, which is capable of cleaving ß-1,4-glycosyl bonds of the sugar substrate in the same way as natural enzyme, cellulase (Figure 4) [49]. 

Since cellulose is biodegradable, another approach is using microbial fuel cells (MFCs). This system uses microorganism(s) to hydrolyze cellulose, then oxidize its metabolites, whichare at the same time electrochemically active. Thus, they act as electron acceptors. Based on this principle, Rismani-Yazdi and colleagues succeeded in using rumen microbiota containing both strict and facultative anaerobes, which effectively hydrolyze cellulose, and conserve energy via anaerobic respiration or fermentation [50]. This cellulose-fed MFCs, consisting of two chambers separated by Ultrex proton-exchange membrane and graphite plates as electrodes gained a maximum power density of 55 mW m^−2^ (1.5 mA, 313 mV) and the current was sustained for over two months with the periodically supplementation of cellulose as electron donor. To improve the power density, Rezaei et al. increased the cathode volume (three times larger), ammonia-treated carbon cloth (type A; E-Tek, United States) with a total surface area of 1.13 cm^2^ was used as anode and five two strands of 15-cm-long carbon fiber were used as cathode [51]. Using this modified U-tube MFC and strain ATCC 13047T, a power density of 5.4 ± 0.3 mW m^−2^ could be achieved with much higher current density (119 ± 2.2 mA m^−2^). There have been other designs from MFCs, such as air-cathode one chamber MFCs or three stacked MFCs (achieved 490 mW m^−2^ (0.5 mA)), which not only simplified the FCs but also increased the power density up to 1070 ± 15 mW m^−2^ [52,53,54]. On the other hand, the use of carbon-based nanomaterials as supporting matrix for catalytic systems in direct cellulose/cellulosic biomass-based fuel cells is still under intense research. This is because (i) there are difficulties in breaking down cellulose, leading to low power density; and (ii) the high-cost of CBNs compared to the cost of the outcome products. However, with recent developments in preparation processes much lower cost of CBNs could be expected, opening better and more economically viable opportunities for further development of direct cellulose fuel cells. 

### 2.2. Starch-Based Fuel Cells

Starch is made of linear/helical amylose and branched amylopectine molecules. It is much simpler to process than cellulose because it is easier to degrade, thus requires relatively less energy to process. It changes from a crystalline to an amorphous structure and becomes soluble in water by simple heating at low temperature. Different from cellulose, starch is one of the main components in many edible biomass sources, thus, less work has been done to develop a device to extract energy directly from starch. Focus of the research therefore has been set on the wastewater produced from the processing of starch-contained biomass. 

Spets et al. proposed a direct fuel cell operating with Pt–Pd as an anodic catalyst and the cathode electrode contained a catalyst loading of 3.15 mg cm^−2^ of Cobalt-meso-tetraphenylporphyrine (CoTPP) in concentration of 18% on carbon and of 17.5 mg cm^−2^ of MnCo_2_O_4_. This direct fuel cell, operating with 10 g L^−1^ of starch in 2 M KOH, although utilized noble metal as catalyst, could not gain more than 1 mA cm^−2^ at 51 °C [55]. The reason for the low current density is low degradability of starch under alkaline conditions, therefore not many active groups that are able to be oxidized on electrode surface are produced. Still, the current density of starch is much higher than that of cellulose, due to a relatively higher solubility of the starch in alkaline electrolyte. Another attempt to increase the performance of direct starch fuel cell was made by Liu et al., utilizing polyoxometalate (POM) solution without any solid metal or metal oxide as catalyst. This liquid-catalyst fuel cell achieved 34 mW cm^−2^ (at 150 mA cm^−2^) and 22 mW cm^−2^ (at 135 mA cm^−2^), respectively, when starch and cellulose were used directly as fuels at 80 °C [56]. However, since the liquid catalyst is mixed with fuels, it is not yet clear how the regeneration of the catalyst could be done for the next load of new fuels. 

The first MFC based on starch was developed by Niessen and colleagues [57]. They specially designed an anode made of platinum covered by poly(tetrafluoroaniline) and living cells of the biocatalysts *Clostridium butyricum* or *C. beijerinckii*. This MFC (100 mL in volume) attained maximal power density of 1.87 mW cm^−2^, and a corresponding to a current of 4 mA cm^−2^ at a potential of ~473 mV from 10 g L^−1^ of starch. Herrero-Hernandez et al. alternatively used *E. coli* in a mediatorless microbial fuel cell to generate electricity from starch extracted from boiled potatoes. A maximum power density of 502 mW m^−2^ at a current of 0.90 mA was obtained [58]. The improvement of power density in this case was attributed to the use of platinized titanium mesh electrode which was approximately one order of magnitude greater than the maximum output achieved with Pt strip electrodes (66 mW m^−2^). It is obvious that the catalytic surface area in this case plays a big role in improved performance. Although active surface area can be increased significantly with the use of CBNs, such as carbon nanotubes, not much work has been done to utilize CBNs in starch-based fuel cells. 

### 2.3. Alginate Fuel Cells

The usage of biomass as fuel has to deal with a crucially controversial problem before seeing its future. This problem is that its growth is able to consume farmland for food production leading to potential detrimental changesin food supply and therefore increasing food price. To solve this before it becomes an issue, inedible lignocellulosic biomass materials, marine macroalgae, commonly known as “seaweed” stands out as a prominent candidate because they require no fresh water, fertilizer, or land and do not interfere with the human food chain [59]. Energy extraction process from macroalgae can be divided into two stages: (i) release sugars inside the algae cell walls, which is composed mainly of alignate; mannitol and glucan and then (ii) use algae-derived sugars as fuels for energy conversion devices such as fuel cells. Current studies mostly focus on finding an appropriate microbial platform that converts these sugars into ethanol, which is then used as a fuel in combustion engines and/or direct fuel cell systems [60]. Despite the successful engineered microbe systems, which can almost completely degrade glucan and mannitol, the straightforward degradation of alginate remains a hurdle. The first attempt was carried out with gold sheet as anodic electrode, similar to the above discussed direct cellulose fuel cells. However, the power density was low (25 mW m^−2^ at 220 mV and 110 mA m^−2^) [61]. Gold as anodic catalyst can attain high open circuit voltage (620 mV) but the potential steeply decreases with increasing current density, due to insufficient alginate active adsorption on the Au surface and thus results in high internal resistance. To increase the active surface area and mass transfer of substrate, gold nanoparticles were synthesized and then drop casted on functionalized multi-walled carbon nanotubes, leading to 2.1 times higher power density and much lower internal resistance [61]. Although it is still immature, the electrochemical oxidation of alginate in CBNs-based fuel cells has been shown to result in oxidized alginate that is usable for cell and tissue engineering, thus opens a chance for simultaneous production of energy and feedstock materials from inedible biomass. 

## 3. Indirect Energy Conversion Challenges and Applications of CBNs

As discussed above, the energy conversion and therefore power density from lignocellulosic biomass is extremely small for practical applications, although it requires less treatment steps and energy loss during degradation process. Researchers therefore focus on the potentially higher power density generating fuel cells from biomass-derived and smaller molecules such as monosaccharides, ethanol and hydrogen. From these studies, CBNs have been successfully utilized as supporting materials for catalysts, contributing to the improvements of power density and low cost of expected fuel cells as commercial products. 

### 3.1. Monosaccharide Fuel Cells

Glucose, the most well known monosaccharide, is attractive as a fuel for fuel cells, not for energy production applications but for medical applications such as cardiac pacemakers, artificial hearts and glucose sensors [62]. This research trend is still growing, and most of the work has been devoted to the development of catalytic systems that can work in physiological conditions. The power density in this type of fuel cell is not a target for the research, but the stability of the catalysts in implants environment, either noble metal-based or enzymatic types [63]. To date, even pure noble metals suffer from absorbed poisons and intermediates, resulting in performance degradation [64]. The same problem appears in the case of monosaccharide-fed fuel cells as energy conversion devices in addition to the high cost of catalytic materials versus energy proficiency. In order to solve these problems, CBNs, mainly functionalized carbon nanotubes and graphene-based materials were used as the catalytic backbone for noble nanoparticles to (i) increase the active surface area; (ii) decrease noble metal loading; leading to lower cost and (iii) increase stability of catalyst by fine-tuning catalytic mechanism [65]. 

**Figure 5:**
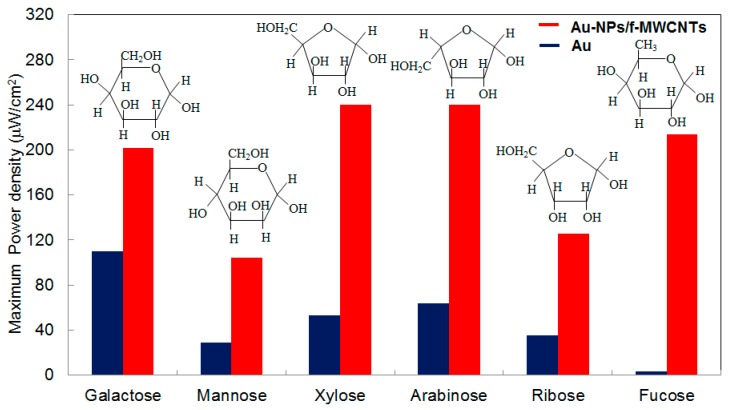
Power density of monosaccharide-based fuel cells using AuNPs/fMWCNTs as anodic catalyst in comparison with bare Au electrode. Reprinted with permission from ref. [66]. Copyright 2011, Elservier.

**Figure 6:**
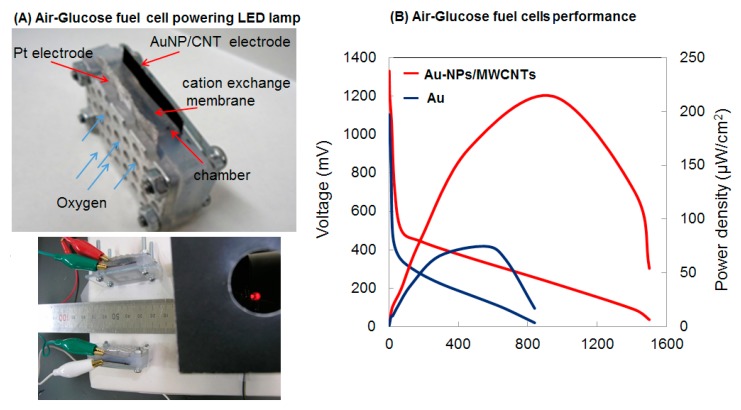
(**A**) Air-glucose fuel cell prototype and (**B**) its performance in terms of polarization and power density. Glucose concentration was 0.4 M in 0.3 M NaOH and 0.1 M PBS; temperature was 25 °C. The working voltage of the LED lamp was 2 V. Reprinted with permission from ref. [66]. Copyright 2011, Elsevier.

It has been shown that the functionalized multi-walled carbon nanotubes (fMWCNTs), which contain a lot of –COOH and –OH groups, are hydrophilic and prevent the aggregation of nanoparticles during activation and thermal treatment processes, thus maintain high total active surface area [66,67]. The use of fMWCNTs as supporting matrix resulted in higher catalytic performance, indicated by ~150–200 mV negative shifts of oxidation peaks compared to that from bare gold electrodes, meaning that relatively less activation energy is needed to overcome. Furthermore, different monosaccharides exhibit different oxidation peaks in terms of position, shape, and current intensity [66]. Since the structures of these monosaccharides differ only at the orientation of –OH groups on C2 and C3, the different oxidation potentials suggest that the specific structural attachments of intermediate complexes on gold nanoparticles decorated on fMWCNTs (AuNPs/f-MWCNTs) induce different activated energies for further oxidization. By dispersing AuNPs on fMWCNTs, the gold surface is much more active and respond more sensitively to the change in –OH orientation, leading to higher oxidation current density than that of bare gold electrode. Consequently, the power density generated from AuNPs/fMWCNTs-based FCs is more than twice of that from Au-based FCs (Figure 5). With this approach, two homemade small (2 mL in volume, Figure 6) glucose or fructose fuel cells could run a led lamp that requires a working voltage of 2 V [66,68]. 

Carbon-based nanomaterials are not only excellent supports for noble catalytic metals, but also for enzymatic systems in enzymatic-based FCs (EFCs). Different from the direct oxidation of fuels on catalytic metal surface, the active centers in enzymes are buried and insulated by the protein shell, leading to detrimental effect of electron transfer between the enzyme and the current collector. Based on electron transfer mechanism, EFCs can be divided into two types: Direct electron transfer (DET) and mediator electron transfer (MET). DET requires both proper orientation of an enzyme, as well as the distance between the active center as electron donor and the electrode as electron acceptor. This distance has to be within ~2 nm. Since the electron transfer rate depends exponentially on this distance, it is commonly believed that MET which uses mediator cofactor to shuttle electrons from enzyme to electrode, might be the better choice of the two. However, CBNs have altered this mindset. CNTs, graphene-based materials and most recently carbon nanodots (CNDs) have been used to create direct “wiring” systems since their size is quite close to that of enzymes [69,70,71,72,73,74]. For instance, Ivnitski et al. demonstrated direct glucose oxidation on glucose oxidase (GOx) immobilized on CNT-modified porous bioanode as well as the direct reduction of oxygen with laccase [70]. Although direct enzymatic oxidation of glucose is still highly debated and the mechanism is not yet clearly proven, the fact that glucose oxidase is able to simultaneously undergo DET with the electrode and to retain its catalytic activity has been confirmed by the cyclic voltammetry study of the GOx immobilized on the surface of CNTs modified electrodes in the absence and in the presence of glucose. This GOx–CNTs-based membraneless FC achieved an open circuit potential of ~400 mV vs Ag/AgCl. Graphene was used in the same way as CNTs to co-immobilize with GOx on anode and bilirubin oxidase (BOD) for cathodic catalyst, resulting in a maximum power density of about 24.3 ± 4 μW cm^−2^. This is nearly three times greater than that of the SWCNT-based biofuel cell [71], and the performance of the graphene biofuel cell lasted for a week. Instead of graphene sheets, Zheng and colleagues made use of nanographene platelets (NGPs) to immobilize GOx and BOD, and achieved a maximum power density of 57.8 μW cm^−2^ [73]. A higher open circuit potential (0.93 V) and a maximum power density of 40.8 μW cm^−2^ (at 0.41V) were achieved recently by Zhao and colleagues with DET of GOx and BOD at Carbon Nanodots (CNDs) electrodes [72]. This CND-based glucose FCs surpassed the power density could be achieved from MET-type BFCs in which an oxygen independent Pyrroloquinoline Quinone (PQQ)–GDH–MWCNT-electrode coupled with a BOD–MWCNT-electrode were used (23 μW cm^−2^). The success of CBNs and enzymatic systems has meant that the use of noble metals could be avoided, thus reducing significantly the cost of the final product. However, when the power density is the target, the composite of CBN with noble metals increases power density even more. By anchoring AuNPs on reduced graphene oxide, followed by electrochemical polymerization of neutral red (RGO/AuNPs/PNR) before drop-casting GOx, the power density gained from this electrode was increased up to 176 μW cm^−2^ [74]. Zebda and colleagues, thus far, have reported the highest power density. In their work, glucose oxidase and laccase were mechanically compressed into CNT disks [75]. This led to homogenous dispersion of biomolecules within the CNT supporting matrix. However, in the presence of oxygen within the non-wired GOx matrix, a possible formation of hydrogen peroxide may degrade GOx performance. To overcome this problem, catalase was added to the GOx–NT mixture before compression to decompose H_2_O_2_. This improved mediatorless glucose biofuel cell generated a high power density up to 1.3 mW cm^−2^ and achieved an open circuit voltage of 0.95 V. Moreover, the fuel cell performance remained stable for a month and delivered 1 mW cm^−2^ power density under physiological conditions (5 × 10^−3^ M glucose, pH 7). 

When using for MET-based glucose fuel cell, CNTs-based FCs achieved up to 740 μW cm^−2^ in the presence of 0.015 M glucose, which is tenfold higher than that of the same catalytic system using traditional carbon fiber supporting matrix [76]. Kim et al. recently reported a simple method for simultaneous CNT dispersion and sequential enzyme adsorption, precipitation, and crosslinking that resulted in 7.5 times higher power output than that from the covalently-attached GOx on acid-treated CNTs and GOx activity remained stable for 270 days [77].

### 3.2. Bio-Ethanol Fuel Cell

**Figure 7:**
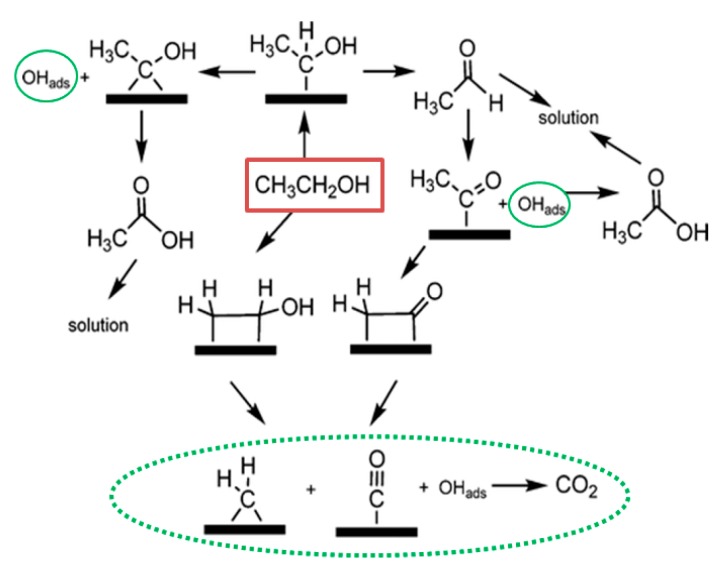
Ethanol oxidation reaction pathway.

Bioethanol is now practically being used as a blend or fossil petrol substitute. For example, in the USA the use of ethanol as a blended component has increased dramatically from about 1.7 billion gallons in 2001 to about 14.4 billion in 2016 [78]. However, the “food-versus-fuel” issue is still problematic because ethanol is mostly produced from edible feedstock such as corn. Researchers are therefore, targeting non-edible feedstock including waste from agriculture and forestry and brown macroalgae to produce second generation of bioethanol [79,80]. This is done by fermentation followed by distillation and drying process. Burning ethanol in the way it has been done with fossil fuel, however, is not the best and “green” way to utilize energy from ethanol. Direct ethanol fuel cells (DEFCs) have been reviewed in detail by Kamarudin et al. and Badwal et al., revealing the current challenges, developments and applications of DEFCs [81,82]. However, there is lack of information about CBNs’ studies. An effective yet durable catalytic system for ethanol oxidation reaction in PEMFC has been the target of research and could be divided into two different categories. First, the focus was on understanding the catalytic mechanisms; increasing the durability and decreasing the cost of noble-based materials by making alloys such as Sn/SnO_2_/Pt. One of the main advantages of using these non-noble metal/metal oxide is to create –OH absorption, thus enhancing the removal of CO_ad_ and other poisonous species out of Pt surface (Figure 7). In some special cases, such as Pt–S–-Rh/C catalysts, it has been reported that C–C cleavage could be achieved, producing CO_2_ as final product instead of acetic acid [83]. 

**Figure 8:**
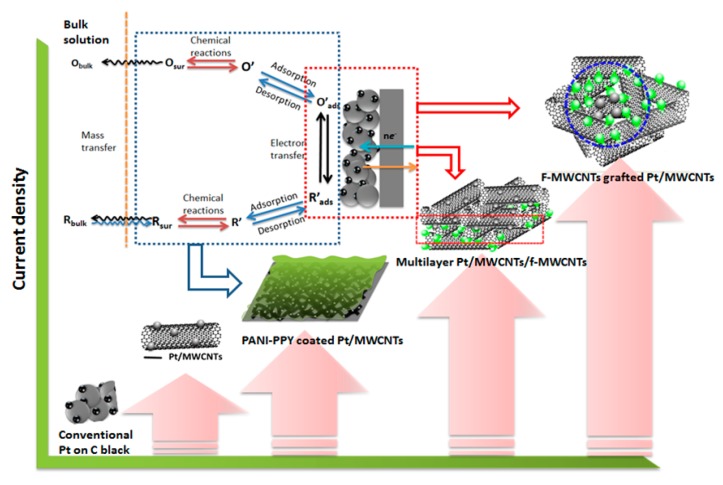
Development of anodic catalytic materials for enhanced direct ethanol fuel cells using co-assembled PANI–PPY and functionalized carbon nanotubes matrices.

The second and recently explored type of research is the use of CBNs as supporting matrix which can act much less the same as the alloyed systems in terms of detoxification; or be combined with alloyed catalyst to further increase the electrocatalytic activities toward complete ethanol oxidation [84,85,86,87,88,89,90,91,92,93,94]. Goel and Basu reported a comparative study of CBNs-supported Pt–Ru and revealed that the maximum power density to be in the order Pt–Ru/MCN (mesoporous carbon nitride) > Pt–Ru/t-MWCNTs > Pt–Ru/MWCNTs > Pt–Ru/Vulcan-XC [84]. The highest power density of 61.1 mW cm^−2^ at 100 °C, 1 bar pressure with catalyst loading of 2 mg cm^−2^ using 2 M ethanol feed could be achieved. Our group alternatively fabricated conducting polymers composited with low-loading Pt nanoparticles decorated on functionalized carbon nanotubes (Pt/MWCNTs) and examined catalytic activities toward ethanol oxidation reaction [88]. In native form, polyaniline (PANI) and polypyrrole (PPY) individually deposited on Pt/MWCNTs exhibited lower catalytic activities over ethanol oxidation than the bare Pt/MWCNTs. However, the co-assembled PANI–PPY deposited on Pt/MWCNTs significantly enhanced both the reaction kinetics and stability of catalysts compared to the one without conducting polymers as revealed by electrochemical measurements. These enhancements were attributed first to the active interface between Pt nanoparticles and conducting polymers, and second to the interaction between PANI and PPY and their contribution to the reaction on Pt surface. Based on the understanding from co-assembled PANI–PPY, it was suggested that the functional groups on polymer backbone may have a crucial effect during the ethanol oxidation reaction. To explore their roles, MWCNTs were used as a highly conductive and chemically inert backbone to attach the desired functional groups. –COOH and –NH_2_ groups were chosen since both of them could create the dissociative adsorption with water molecules via H-bonding and simultaneously induce charged non-covalent interactions [89,90]. The results showed that the COOH–MWCNTs supported Pt/MWCNTs enhanced the ethanol oxidation reaction kinetics by about three times more than NH_2_––MWCNTs and the bare Pt/MWCNTs, in terms of oxidation current density and stability. The results indicated that functional groups functionalized on supporting matrix for catalytic enhancement on Pt active sites play a very important role. In addition, they clearly showed the differences in the effect from specific functional groups depending on their chemical species. We also studied the various structural assemblies between f–MWCNTs and Pt/MWCNTs and how they affected kinetic reactions (Figure 8). 

Instead of CNTs, graphene oxide was exploited and successfully applied as matrix to attach noble metals such as Pt and Pd [85]. Kumar et al. reported a simple procedure to synthesize large-scale perforated graphene oxide nanosheets-Pd hybrids (Pd–FP–rGONSs) using microwave irradiation. The prepared Pd–FP–rGONSs were composed of a large amount of structural surface defects which created porosity inside the nanosheets, leading to lower charge transfer resistance and negative oxidation peak shifts. As a result, Pd–FP–rGONSs performed much better in terms of current density (10.2 mA cm^−2^) than Pd/MWCNTs (0.4 mA cm^−2^) at the same initial ethanol concentration. To date, however, there is no published work yet on the final prototype and its power density of DEFCs, which use graphene oxide-based catalyst. 

Similar to other types of biofuel cells, ethanol biofuel cells can be divided into two main types: Enzyme-based and microbial-based FCs. The former one uses alcohol dehydrogenase to catalyze the oxidation of ethanol, and faces similar electron transfer problems as the above-discussed enzyme-based glucose BFCs. Additionally, the use of NAD-dependent enzymes such as ADH needs to regenerate NAD^+^ species and restore the enzyme cycle. Last but not least is the high over potential of NADH (more than 1 V) and the passivation of the electrode surface such as noble metals. To overcome this problem, the electrode surface was modified with MWCNTs to mediate the electrocatalytic oxidation of NAD-dependent enzymes [91], decreasing the high overvoltage for NADH oxidation of 0.16 V [92]. Besides decreasing the over voltage of NAD^+^/NADH system, modification of electrode by CNTs has been proved to enhance the efficiency of enzyme in power density and electron transfer kinetics [93]. Gouranlou et al. recently reported an ethanol–oxygen biofuel cell design in which PDDA/ADH/PDDA/HOOC–MWCNTs/PMG/GC and PDDA/Lac/PDDA/HOOC-MWCNTs/PMG/Gr operated as bioanode and biocathode, respectively (PDDA and PMG stand for polydiallyldimethylammonium chloride and polymethylene green, respectively). HOOC–MWCNTs beside PDDA has been proved to provide a suitable microenvironment to preserve the activity of ADH and laccase [80]. In the optimized condition, this BFC produced a power density of 3.98 mW cm^−2^ with the OCP of 0.504V. With the use of COOH–MWCNTs as cathodic catalyst, the power density was increased much more than that of traditional Pt/C electrode, which could achieve only 1.713 mW cm^−2^ and OCP of 0.281 V [94]. 

### 3.3. Bio-Hydrogen Fuel Cell

Among all types of fuel cells, the hydrogen fuel cell is the most successful one in terms of power density, hence high possibilities for wide application. Although a hydrogen fuel cell is a zero emission energy device, it is still not a true “green” option since the hydrogen production is mostly from large-scale natural gas reforming processes. Furthermore, storage and transportation issues still present problems for the commercialization of hydrogen as a fuel [95]. To solve the production problem, much works have been devoted to developing bio-H_2_ production technologies based on either “classical” metabolically engineered microorganism or mixed bacterial consortia under controlled nutrient condition to modify gene expression towards increased H_2_ production [96,97,98,99,100]. These approaches enable H_2_ production from not only pure biomass-based fuel such as glucose but also raw and complex materials such as starch or wastewater. On the other hand, direct connection of biohydrogen reactors and onsite energy conversion devices could be an answer to storage and transport problems. Most recently, Wenzel and colleagues reported a MFC coupled to biohydrogen reactor as a feasible technology to increase energy yield from cheese whey. Effluent from dark fermentation of cheese whey was successfully used to produce a maximal power density of 439 mW m^−2^ [100]. 

**Figure 9:**
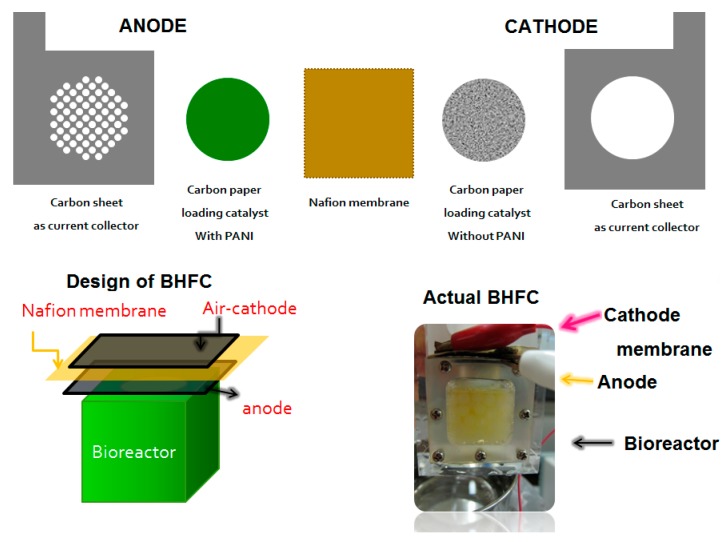
Fuel cell components and the assembly of hydrogen fuel cell with bioreactor.

Unfortunately, hydrogen production processes from biomass often involve the use of microorganisms, which require voluminous bioreactors to ensure a sufficient production rate; and a filter to remove unwanted gases. Researchers so far have tried to integrate the whole fermentation system and a proton-exchange-membrane fuel cell (PEMFC) for electricity generation [101,102,103,104]. Still much extra energy is needed to maintain the bioreactor, filter, and the pumping system. In our group, we demonstrated a compact single chamber-based fuel cell that changes the anode chamber of the hydrogen PEMFC to an anaerobic fermentation bioreactor (Figure 9). This combination greatly reduced the external energy needed for maintaining the bioreactor, condenser and/or filter, for storing gases under high pressure (optional) and for the humidifier before pumping the hydrogen into the PEMFC. Consequently, the total useful energy from the whole system increased [105,106]. 

However, due to direct attachment of bioreactor to anode, unwanted gases and microorganisms in the anode chamber could enter the pores of the electrodes via water vapor, resulting in a significant reduction of gas dispersion inside the catalyst layers and lower catalytic activity. To address this problem, doped polyaniline (PANI) nanofibre-based composites with Pt/fMWNTs were used instead of traditional PtNPs on carbon black to achieve much higher conductivity, higher catalytic activity under humid condition, more access to the gases resulting from a three-dimensional structure with more active sites, and more resistance to corrosion. The hybrid structure was made in two ways: multilayered and core-shell. The maximum power density from the former (733 mW m^−2^ of PANI/Pt/fMWNTs) was more than twice compared to that of the later one (352.75 mW m^−2^ of Pt/fMWNTs@PANI). The enhanced performance in case of multilayer structure was made possible by the active contact between the PANI nanofiber layer and Pt/fMWNTs that facilitates selective hydrogen absorption and increases conductivity at high humidity [106]. The power density of this biohydrogen fuel cell was much higher than the MFCs using CNTs/PANI composite as anode material (42 mW m^−2^) in Qiao et al. [107], and comparable with other single chambered, glucose-fed mediatorless MFCs using *E. coli* or mixed anaerobic consortia [108,109,110,111,112]. 

Another approach is using H_2_/O_2_ biofuel cells, in which hydrogenase is used as anodic catalyst and multicopper oxidase as cathodic catalyst. It is estimated that the maximum current density would be 1 mA cm^−2^, when the enzyme forms an electrocatalytically active monolayer on a planar electrode. Thus, three-dimensionally structured CBNs have been explored to increase enzyme loading and efficient orientation of enzyme towards enhanced electron transfer rate. The power density of FCs has reached 8 mW cm^−2^ and the current density is up to 10 mA cm^−2^ [113,114,115,116,117]. The fuel in these cases was mostly pure H_2_ or mixture of pure H_2_ and O_2_ or air, which is different from biogas generated directly from biohydrogen reactors, and therefore out of our scope in this mini review. Nevertheless the development of H_2_/O_2_ biofuel cells towards the use of biomass-generated hydrogen could be a promising future study. Details about recent studies on CBNs in H_2_/O_2_ biofuel cells were thoroughly reported in a review article by Mazurenko et al [118]. 

## 4. Concluding Remarks

It is obvious that the future of biofuels as alternative to fossil fuel requires technological development of efficient but low-cost biofuel-based energy conversion devices. Biofuel-fed fuel cell, which can work at low temperature, are non-toxic, have low carbon emissions, and utilize a wide range of biofuel sources in both pure and non-pure forms (e.g., from wastewater), is therefore a promising technology. However, there are still many challenges that need to be overcome before reaching practical and commercial states. As presented above, although the direct biofuel-fed fuel cells generate electricity directly from main biomass components such as cellulose, starch, and alginate, the power density is too low, thus the only possible application is coupling with the wastewater treatment process to extract energy simultaneously with the degradation of biomass as organic wastes. Along with the development of breakdown technology, many more efforts have been devoted to develop fuel cells that indirectly extract energy from biomass i.e., from secondary fuels such as monosaccharides, bioethanol, and biohydrogen. So far, the power density of such biofuel-fed fuel cells could reach the demand of portable devices but fuel cell fabrication cost and its durability are still the main hurdles. For both direct and indirect biofuel-fed fuel cells, the main challenges are high cost yet low durability of catalytic systems, resulting in limited fuel cell performance. To replace expensive noble metal-based catalysts, carbon-based nanomaterials have been explored and contributed significantly to the improvement of fuel cell performance. When used as supporting matrix, carbon-based nanomaterials such as graphene, CNTs, and their functionalized materials create a 3-D architecture for anchoring metal nanoparticles/microorganism/enzyme with much higher surface area to volume ratio, enhanced electrolyte diffusion and simultaneously act as electron transfer network. Furthermore, functionalized carbon-based nanomaterials have been proven to be involved in removing toxic intermediates on the embedded nanoparticles in case of metallic catalysts and enhanced immobilization and stabilization of enzymatic systems, resulting in improvement of catalytic durability. As a result of using CBNs, the power density of biofuel-fed FCs increased from nW to mW cm^−2^, open circuit potential increased to more than 1 V (fucose and glucose FCs) and life-time of fuel cell performance was significantly extended. Based on these achievements, future research trends could be seen targeting (i) deeper understanding and optimizing current successful CBNs for each type of biofuel-fed FCs; and (ii) discovery of new materials such as composites of well-established and/or entirely new metal-free CBNs towards low-cost but long-term large scale practical applications.

## Figures and Tables

**Table 1:** Summary of carbon-based nanomaterials (CBNs) recent achievements in microbial fuel cells (MFCs) towards enhanced cathode performance.

Reactor Type	Anodic Supporting Materials	Cathode Materials	Power Density (mW m^−2^)	Comparision to Pt/C or Bare Materials	Ref.
250 mL double chambers	Graphite granule	Pt/C	1470 ± 10	-	[35]
250mL double chambers	Graphite granule	Co_3_O_4_/N doped graphene	1340 ± 10	Comparable to Pt/C	[35]
6 mL single chamber	Carbon paper	Co/Fe/N/CNTs	751	1.5 times > Pt/C	[36]
80 mL double chamber	Carbon fiber	N-doped CNTs on C cloth	1600 ± 50	12.8% > Pt/C	[37]
Single chamber	Carbon felts	Fe–N-functionalized graphene	1149.8	2.1 times > Pt/C	[38]
Single chamber	Carbon cloth	Graphene supported Pt–Co	1378	Comparable to Pt/C	[39]
Sediment MFCs	Graphite felt	Polyaniline-Graphene nanosheets	99	116 times > Graphite	[40]
Double chamber	-	N/S co-doped carbon nanosheets	1500	Comparable to Pt/C	[41]
15 mL sediment single chamber	MWNT coating Graphite	MWNT coating Graphite	214.7 ± 9.9	1.6 times higher than that of bare graphite	[42]
Single chamber	Carbon cloth	Graphene/poly(3,4-ethylenedioxythiophene)/Fe_3_O_4_	3525	8.2 times > Carbon cloth	[43]
Double chamber	Carbon brush	N-doped Graphene/CoNi alloy encased within bamboo-like CNTs	2000	Comparable to Pt/C	[44]
210 mL double chamber	Carbon paper	CNTs/Polypyrrole	113.5	Comparable to Pt/C	[45]
Single chamber	Graphite fiber brush	Ag/Fe/N/C	1791	1.5 times > Pt/C	[46]
20 mL single chamber	N-Ni-carbon nanofibers	N-doped polymer-Ni–carbon nanofibers	1690 ± 30	-	[47]
1.5 L single chamber	Carbon fiber	Co-doped Carbon nanofibers	92	Stable more than 6000 h	[48]
